# Optimizing hydroponic culture media and NO_3_^−^/NH_4_^+^ ratio for improving essential oil compositions of purple coneflower (*Echinacea purpurea* L.)

**DOI:** 10.1038/s41598-021-87391-9

**Published:** 2021-04-13

**Authors:** Fatemeh Ahmadi, Abbas Samadi, Ebrahim Sepehr, Amir Rahimi, Sergey Shabala

**Affiliations:** 1 Department of Soil Science, Faculty of Agriculture, Urmia University, Urmia, Iran; 2 Department of Plant Production and Genetics, Faculty of Agriculture, Urmia University, Urmia, Iran; 3 Tasmanian Institute of Agriculture, University of Tasmania, Hobart, TAS 7001 Australia

**Keywords:** Plant sciences, Medical research

## Abstract

Medicinal plants represent a valuable commodity due to beneficial effects of their natural products on human health, prompting a need for finding a way to optimize/increase their production. In this study, a novel growing media with various perlite particle size and its mixture with peat moss was tested for hydroponic-based production of *Echinacea purpurea* medicinal plant under greenhouse conditions. The plant growth parameters such as plant height, total fresh leave weight, fresh root weight, total biomass, total chlorophyll, leaf area, and essential oil compositions were assessed. Perlite particle size in the growing media was varied from very coarse (more than 2 mm) to very fine (less than 0.5 mm), and the ratio between perlite and peat moss varied from 50:50 v/v to 30:70 v/v. In addition, two nitrate (NO_3_^−^) to ammonium (NH_4_^+^) ratios (90:10 and 70:30) were tested for each growing media. The medium containing very fine-grade perlite and 50:50 v/v perlite to peat moss ratio was found to be most optimal and beneficial for *E. purpurea* performance, resulting in maximal plant height, fresh and dry weight, leaf surface area, and chlorophyll content. It was also found that an increase in NO_3_^−^/NH_4_^+^ ratio caused a significant increase in plant growth parameters and increase the plant essential oil content. The major terpene hydrocarbons found in extract of *E. purpurea* with the best growth parameters were germacrene D (51%), myrcene (15%), α-pinene (12%), β-caryophyllene (11%), and 1-Pentadecene (4.4%), respectively. The percentages of these terpene hydrocarbons were increased by increasing of NO_3_^−^/NH_4_^+^ ratio. It can be concluded that decreasing the perlite particle size and increasing the NO_3_^−^/NH_4_^+^ ratio increased the plant growth parameters and essential oil compositions in *E. purpurea*.

## Introduction

Medicinal plants and their beneficial effects on human health are well known in various cultures for centuries^1^. *Echinacea* is a medicinal plant that belongs to the family of Asteracea/Compositae and is native to much of the United States^2,3^. The most popular species of the plant in medicine are *E. purpurea*, *E. angustifolia*, and *E. pallida*. The species has a black and pungent root and purple coneshape flowering head^4^. All parts of the *E. purpurea* species, especially root and coneflower, are rich in useful medicinal compounds, prompting significant attention of researchers to this species^5,6^.

Using of *E. purpurea* essential oil in medicinal, cosmetic, and food industries is common in all over the world^7^. The effect of *E. purpurea* essential oils on antimicrobial properties has been proven in previous studies^8^. Also accepted is the role of some constituents of the essential oil of *E. purpurea*, including α-phellandrene, myrcene, limonene, α-pinene, β-pinene a, δ-cadinene, germacrene D, and β-caryophllyene, as antifungal, antiviral and antibacterial agents^9,10^. Extracts of essential oil obtained from *E. purpurea* are efficient in pest control and could regulate insect population at different life stages^11^. Numerous studies have been focused on prominent insecticidal influence of *E. purpurea* essential oil compositions and found the better influence of them in comparing with chemicals or a potential source of insecticides^11^. The antibacterial activity of *E.purpurea* essential oil is also reported against different food pathogens and bacteria in food industry^12,13^.

While the industrial application of *E. purpurea* essential oils is well established, several factors such as weather changes, plant growth stage^14^, and method of cultivation may influence both the composition and production of *E. purpurea* essential oil^15^. The open field cultivation of *E. purpurea* has some significant limitations such as crop inconsistency, seed dormancy^16^, water stress regims^17^, microbes, heavy metal ions and other pollutants^2^ and loss of wild germplasm, that affect the different chemical composition of the plant extract. The above limitations have prompted a shift towards plant production under greenhouse conditions, especially in hydroponic (or soilless) culture systems^18^. Growing in a greenhouse also offer an additional advantages of more effective control of plant nutrition^19,20^.

Different hydroponic cultivation methods, such as artificial substrate media, water culture, and nutrient film techniques have been reported for *E. purpurea* cultivation^18,21^. However, using artificial substrates in the hydroponic cultivation system reduces the cost of establishing advanced hydroponic cultivation systems and also enables the farmer to make a practical use of it by using commonly raw materials such as cocopeat, sand, and vermiculite as an initial plant growing media^22^. Nevertheless, different inorganic products such as peat moss, perlite, mixed materials, etc. are fully or partially used instead of initial substrates due to their useful physical properties. The particle size of substrates is a critical factor in air and water-holding capacity, root distribution, and plant growth, which are different based on their origin and preparation conditions. A high volume of roots can concentrate at the top portion of the container includes low aeration and high water-holding capacity^22^.

In addition to the importance of substrates properties in the hydroponic culture system, attention to the chemical composition of nutrient solution is important^22^. In terms of chemical composition of nutrient solutions, two major inorganic forms of nitrogen (N), the NH_4_^+^ and the NO_3_^−^, can differentially impact the various plant properties, based on the plant species^18^. Although the assimilation and metabolism of NH_4_^+^ form require less energy than that of NO_3_^−^ in plants, the majority of plant species grow better on NO_3_^−^ since NH_4_^+^ is toxic for plants and a few species grow well if NH_4_^+^ is the only source of N^4^. The plant species and environmental conditions are two critical factors that affect the optimum NO_3_^−^/NH_4_^+^ ratio^23^. It has been reported that different N application rates could affect the essential oil compositions of peppermints (*Mentha piperita* L.)^24^. Previous researches also demonstrated that the inorganic N application rate and sources could affect the essential oil content of sweet basil (*Ocimum basilicum* L.) and forage maize (*Zea mays* L.)^25^.

Although many researches have been performed on hydroponic culture of *E. purpurea*, but the use of culture media with different perlite particle sizes, different NO_3_^−^/NH_4_^+^ ratios, and their effects on essential oil compositions of *E. purpurea* has been assessed for the first time in this study. So the main goal of this study was to investigate the growth parameters and essential oil compositions of *E. purpurea* growing in new hydroponic culture media with various perlite particle sizes and different NO_3_^−^/NH_4_^+^ ratios.

## Materials and methods

### Growth conditions

The experiment was performed in a commercial greenhouse at Urmia University, West Azerbaijan, Iran. The air temperature was 22/18 °C (day/night) and the humidity ranged from 70 to 80%. The maximum photosynthetic photon flux density (PPFD) fluctuated from 550 to 750 μmol m^−2^ s^−1^ inside the greenhouse. The *E. purpurea* seeds were purchased from Iranian private joint-stock company, Pakan Bazr Esfahan (www. Pakanbazr.com). The seeds were sowed in plastic cups filled with a mixture of perlite and peat moss substrates as a medium to initiate germination. Irrigation was performed based on greenhouse conditions regularly. Seedlings (with four real leaves) were translocated to experimental plastic pots (2.5 L) containing a different ratios of perlite and peat moss as artificial substrates (100% perlite, 100% peat moss, 50% (v) perlite + 50% (v) peat moss, 70% (v) perlite + 30% (v) peat moss) with various perlite particle size containing less than 0.5 mm, 0.5–1 mm, 1–1.5 mm, 1.5–2 mm, and more than 2 mm. Chemical concentrations of nutrient solution are shown in Table 1. The pH and electrical conductivity (EC) of the nutrient solution were maintained between 5.7 to 6.2 and 1.0 to 1.5 dS m^−1^, respectively. According to the stage of the plant growth, 0.5 to 3.5 L day^−1^ was used in fertigation system^18^.

**Table 1:** Chemical properties of nutrient solution.

Element	Fertilizer type	Amount
Nitrogen (N)	(NH_4_)_2_SO_4_-KNO_3_-Ca(NO_3_)_2_	15 mM
Phosphorus (P)	H_3_PO_3_	1 mM
Potassium (K)	KNO_3_	6 mM
Calcium (Ca)	Ca(NO_3_)_2_	4 mM
Magnesium (Mg)	MgSO_4_·7H_2_O	2 mM
Sulfur (S)	Sulfate fertilizers	2 mM
Iron (Fe)	Fe-EDTA	50 µM
Manganese (Mn)	Mn SO_4_·H_2_O	9 µM
Copper (Cu)	CuSO_4_·5H_2_O	0.3 µM
Zinc (Zn)	ZnSO_4_·7H_2_O	0.8 µM
Boron (B)	H_3_BO_3_	15 µM
Molybdenum (Mo)	H_24_Mo_7_N_6_O_24_·4H_2_O	0.11 µM

### Sample preparation

Plants were harvested at the end of the flowering stage (eight months). The plants were divided into roots, stems, flower heads, and lower and upper leaves after washing with tap water. Root, flower heads, and leaves samples were dried at 25 ± 1 °C, ground into a fine powder and collected for further analyses^6^.

### Plant growth parameters

The main growth parameters such as plant height (cm), fresh root weight (g plant^−1^), total fresh leave weight (g plant^−1^), total biomass (g plant^−1^), and leaf area (cm^2^) were determined for each plant at the matured stage. The leaf area was measured by using leaf area meter. Chlorophylls a and b were determined using 0.5 g of dry sample, which was homogenized with 10 mL acetone. Homogenized samples were centrifuged at 10,000×g for 15 min at 4 °C^2^. The supernatant was separated, and the absorbance spectra were measured at 400–700 nm. The total chlorophyll was calculated at 645 nm and 663 nm respectively. So that^26^:

1 $${\text{C}} = {2}0.{2}\;{\text{A}}_{{{645}}} - {2}.{35}0\;\;{\text{A}}_{{{663}}}$$

where C is the total chlorophyll contents in mg/L of acetone extract, A_645_, and A_663_ are the absorption of the extract at 645 and 663 nm.

### Extraction of essential oils

The *E. purpurea* plants which shown the best morphological properties (maximum height, dry and wet weight of leaves and roots, and leaf area) were selected for analysis of essential oil. Distilled water was added to 120 g powder samples (root, leaves, and flower head) at a 1:10 (g mL^−1^) ratio. The essential oil was extract based on the distillation procedure using a commercial Clevenger apparatus^27^.

### Analysis of essential oil

The essential oil analysis was performed using gas chromatography (GC) with 30 m × 0.25 mm capillary column coated with 0.25 µm film; carrier gas, helium (He) with a flow rate of 32 cm s^−1^; injector temperature of 260 °C and injection volume 0.2 µL. The programming was carried out from 90 °C for 2 min rising at 7 °C min^−1^ to 180 °C, at 15 °C min^−1^ to 220 °C. Identifications of different components were made by library search program on monoterpenoids and sesquiterpenoids mass spectral database and by comparing retention time with those of reference samples^27^.

### Gas chromatography–mass spectrometry

Gas Chromatography–Mass Spectrometry (GC–MS) spectra were recorded on a Varian-3400 model fitted with a fused silica capillary column (30 m × 0.25 mm i.d.) coated with 0.25 µm film. The GC was run from 60 to 250 °C at a programmed rate of 8 °C min^−1^, hold at 100 °C for 2 min, using He as the carrier gas at a pressure of 1.6 kg cm^−2^ and injector temperature of 250 °C. The GC column was coupled directly to the quadrupole mass spectrometer operated in the electron impact (EI) mode at 70 eV. Mass spectra were recorded at a scan speed of 9 at m/z 700–10.

### Statistical analysis

The statistics was based on the factorial with completely randomize design with three replications. The factors contained different sizes of perlite, including very coarse perlite (more than 2 mm), coarse perlite (1.5–2 mm), medium perlite (1–1.5 mm), fine perlite (0.5–1 mm), and very fine perlite (less than 0.5 mm), two NO_3_^−^/NH_4_^+^ rations (90:10 and 70:30), and a mixture of peat moss with different size of perlite at 50:50 v/v and 30:70 v/v peat moss to perlite ratios and pure peat moss (100% by volume). Data were analyzed using Duncan's multiple range tests at *P* ≤ 0.01, using statistical analysis software (SAS, 9.4; SAS Institute, 2011) statistical program.

### License for the collection of plant specimens

The authors declare that the collection of plant and seed specimens were according to authorized rules.

### Complying with relevant institutional, national, and international guidelines and legislation

The authors declare that all relevant institutional, national, and international guidelines and legislation were respected.

## Results

### Plant growth parameters

**Figure 1:**
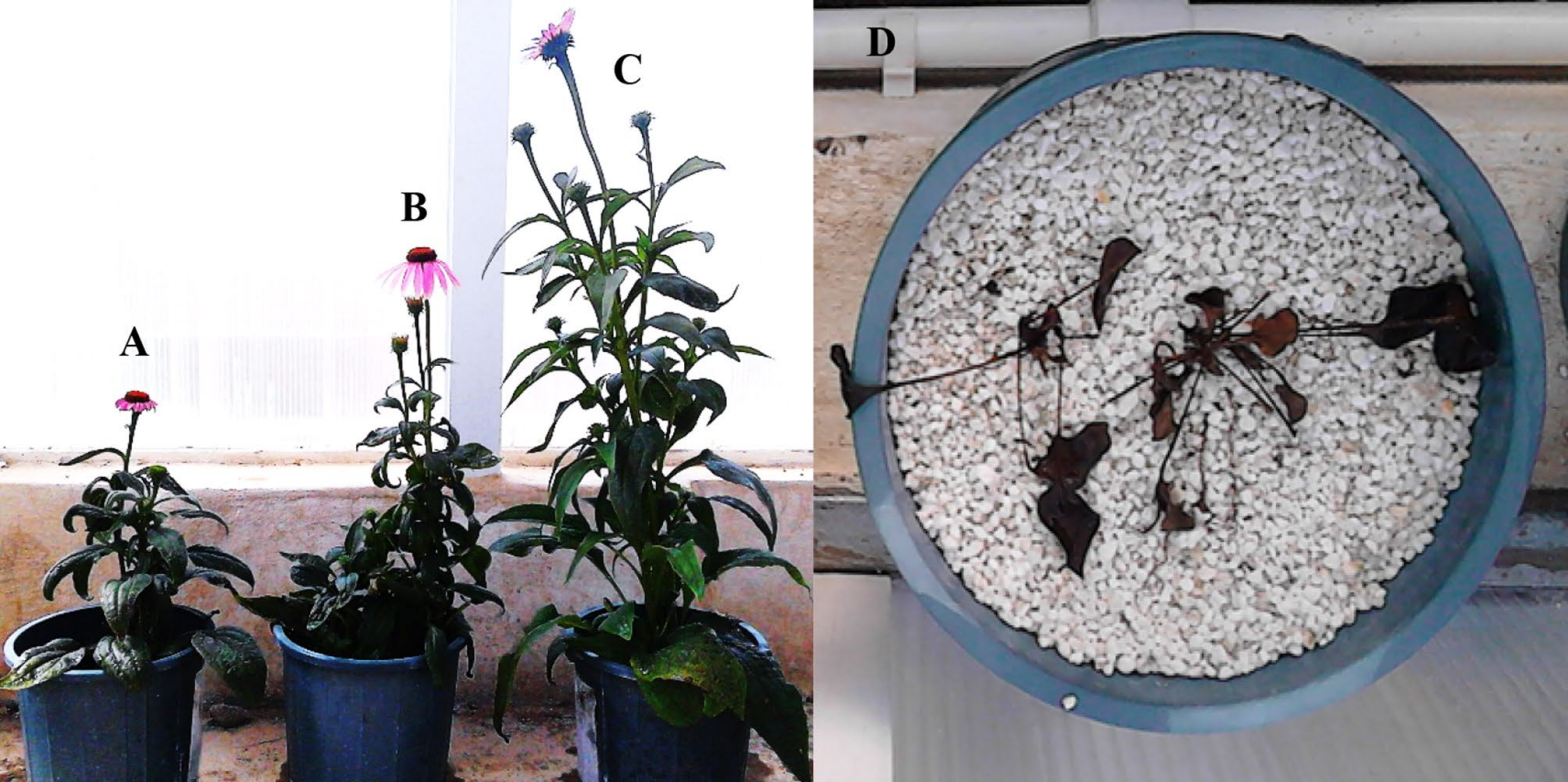
*Echinacea purpurea* grown in (**A**) 100% peat moss, (**B**) 30% peat moss + 70% perlite (< 0.5 mm), (**C**) 50% peat moss + 50% perlite (< 0.5 mm), (**D**) 100% perlite (> 2 mm) culture media, just at 90:10 NO_3_^−^/NH_4_^+^ ratio. (All photos were taken by F. Ahmadi).

**Figure 2:**
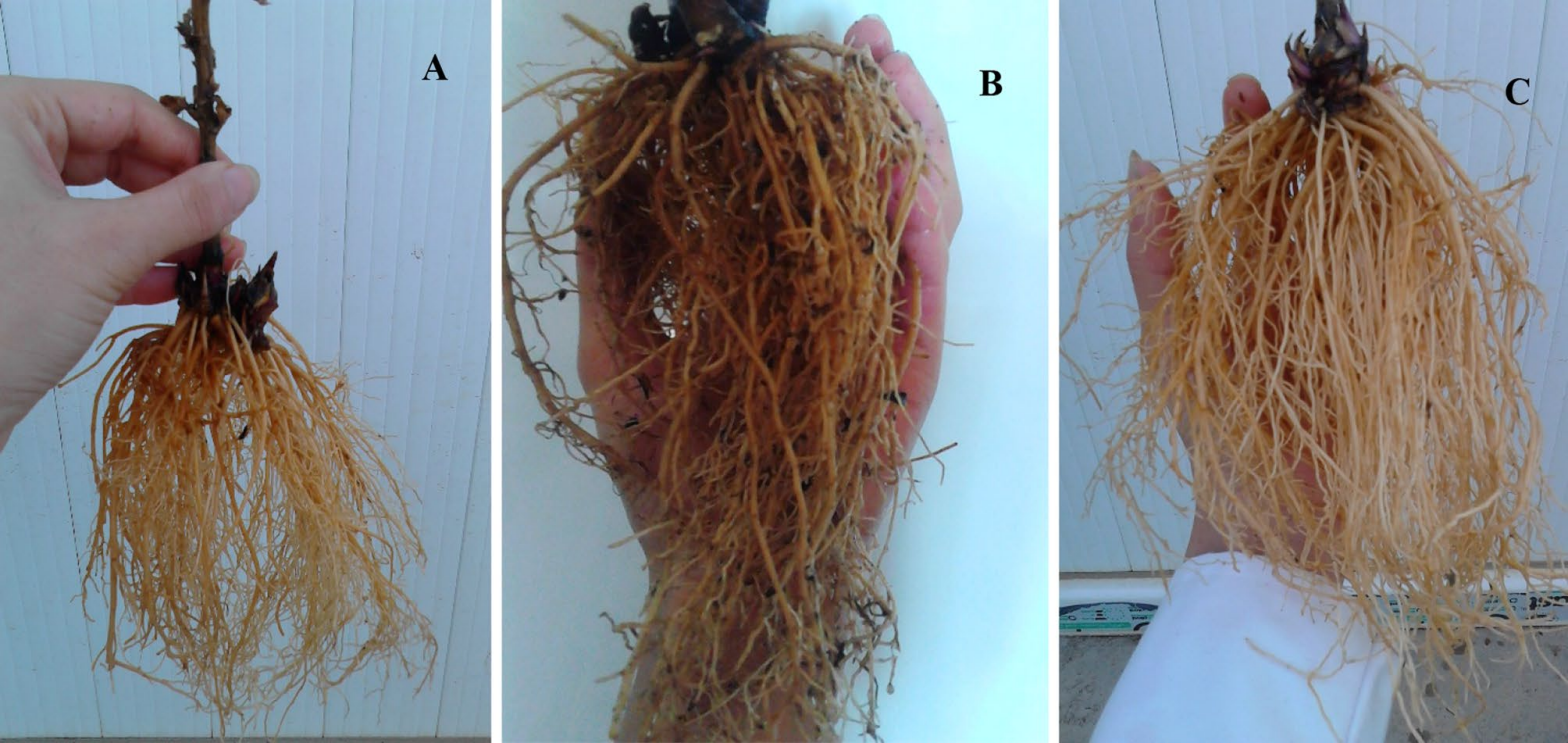
Root morphology of *E. purpurea* grown in (**A**) 100% peat moss, (**B**) 30% peat moss + 70% perlite (< 0.5 mm), (**C**) 50% peat moss + 50% perlite (< 0.5 mm) at 90:10 NO_3_^−^/NH_4_^+^ ratio. The root of *E. purpurea* grown in 100% perlite was very small. (All photos were taken by F. Ahmadi).

Plant growth parameters of *E. purpurea* under different culture media and NO_3_^−^/NH_4_^+^ ratios at the full flowering stage are shown in Table 2 and Figs. 1 and 2.

**Table 2:** Some morphological properties of *E. purpurea* growing on various culture media and NO_3_^−^/NH_4_^+^ ratio at the flowering stage.

Culture media	NO_3_^−^/NH_4_^+^ ratio	Height	Total fresh leave weight	Fresh root weight	Total biomass	Total Chlorophyll	Leaf area
		(cm)	(g plant^−1^)	(g plant^−1^)	(g plant^−1^)	(mg g^−1^ FW)	(cm^2^)
100% Pe (> 2 mm)	90:10	5.3^n^ ± 0.27	1.3 ± 0.13	3.1^s^ ± 0.16	4.4^x^ ± 0.25	5.12 ± 0.11	5 ± 0.41
	70:30	3.2^n^ ± 0.21	1.5 ± 0.11	3.1^s^ ± 0.11	4.1^x^ ± 0.14	3.53 ± 0.032	4 ± 0.35
100% Pt	90:10	55^h^ ± 2.9	10 ± 2.1	20^p^ ± 3.9	30^w^ ± 4.1	8.8 ± 0.15	20 ± 0.25
	70:30	47^jk^ ± 2.1	8.2 ± 1.1	16^r^ ± 2.5	24^y^ ± 3.2	6.6 ± 0.12	15 ± 0.14
50% Pt + 50% Pe (< 0.5 mm)	90:10	105^a^ ± 6.1	40 ± 3.2	75^a^ ± 4.6	116^a^ ± 7.1	18.5 ± 0.11	60 ± 0.35
	70:30	91^d^ ± 4.2	28 ± 1.2	52^d^ ± 3.2	80^d^ ± 4.1	15.1 ± 0.11	51 ± 0.23
50% Pt + 50% Pe (0.5–1 mm)	90:10	98^b^ ± 5.1	27 ± 2.1	53^c^ ± 4.1	81^c^ ± 6.1	16.2 ± 0.13	55 ± 0.15
	70:30	71f. ± 3.2	21 ± 1.1	48f. ± 2.5	70^i^ ± 3.6	13.2 ± 0.11	49 ± 0.11
50% Pt + 50% Pe (1–1.5 mm)	90:10	96^bc^ ± 5.9	26 ± 2.1	50^e^ ± 3.2	76^e^ ± 5.2	14.6 ± 0.16	50 ± 0.15
	70:30	82^e^ ± 3.2	24 ± 1.2	43^i^ ± 1.9	67^jk^ ± 2.4	12.8 ± 0.11	42 ± 0.10
50% Pt + 50% Pe (1.5–2 mm)	90:10	91^d^ ± 4.3	25 ± 1.1	45^h^ ± 2.5	71^hi^ ± 3.6	13.8 ± 0.14	43 ± 0.11
	70:30	71f. ± 2.1	24 ± 1.1	40^k^ ± 1.9	65^lm^ ± 2.4	12.2 ± 0.12	38 ± 0.11
50% Pt + 50% Pe (> 2 mm)	90:10	85^e^ ± 3.3	23 ± 2.1	41^jk^ ± 2.5	64^m^ ± 4.1	13.2 ± 0.12	40 ± 0.14
	70:30	66^g^ ± 2.5	21 ± 1.1	37^l^ ± 2.1	58^p^ ± 3.5	11.5 ± 0.11	32 ± 0.10
30% Pt + 70% Pe < 0.5 mm)	90:10	85.2^e^ ± 2.8	23 ± 1.1	50^e^ ± 3.1	74^g^ ± 4.1	16.1 ± 0.13	42 ± 0.15
	70:30	71.6f. ± 2.1	22 ± 1.1	43^i^ ± 2.4	65^l^ ± 3.6	12.3 ± 0.11	35 ± 0.11
30% Pt + 70% Pe (0.5–1 mm)	90:10	71.1f ± 3.5	21 ± 1.3	47^g^ ± 2.2	68^j^ ± 4.1	13.5 ± 0.12	38 ± 0.15
	70:30	63.9^g^ ± 1.9	20 ± 1.1	37^l^ ± 2.1	58^p^ ± 3.1	10.4 ± 0.11	30 ± 0.11
30% Pt + 70% Pe (1–1.5 mm)	90:10	66.1^g^ ± 2.5	18.8 ± 2.1	42^ij^ ± 2.8	61^no^ ± 4.2	12.9 ± 0.13	32 ± 0.13
	70:30	52.7^hi^ ± 2.1	15.4 ± 1.3	32^n^ ± 1.6	47^s^ ± 2.6	9.4 ± 0.10	25 ± 0.11
30% Pt + 70% Pe (1.5–2 mm)	90:10	55.5^h^ ± 3.2	18.6 ± 1.2	34^m^ ± 2.5	53^q^ ± 3.1	11.3 ± 0.14	28 ± 0.13
	70:30	43.1^kl^ ± 2.1	14.1 ± 1.1	29° ± 1.1	43^tu^ ± 2.1	8.4 ± 0.11	23 ± 0.11
30% Pt + 70% Pe (> 2 mm)	90:10	49.3^ij^ ± 4.2	15.9 ± 2.5	32^n^ ± 2.1	48^rs^ ± 3.6	10.4 ± 0.13	25 ± 0.14
	70:30	35.9^m^ ± 2.1	12.7 ± 1.3	28° ± 1.5	41^v^ ± 2.2	8.3 ± 0.11	19 ± 0.12

Pt: peat moss and Pe: perlite.

The numbers in the parentheses show perlite particle size.

Each value is expressed as mean ± SD (n = 3). Means bearing different letters in the same column are significantly different (*P* ≤ 0.01).

The numbers show as mean ± standard deviation.

The interaction effect of different treatments on total fresh leave weight, chlorophylls a and b, and leaf area was not significant.

Overall, plants grown in the 50% perlite + 50% peat moss medium with perlite particle size less than 0.5 mm and 90:10 NO_3_^−^/NH_4_^+^ ratio had the highest height (mean 105 cm) (Fig. 1), fresh leave weight (mean 30 g plant^−1^), fresh root weight (mean 65 g plant^−1^) (Fig. 2), total biomass (mean 96 g plant^−1^), and leaf area (mean 60 cm^2^). Decreasing perlite percentage of culture media and perlite particle size improved all the morphological properties (Table 2). There were significant differences in the plant morphological properties at different NO_3_^−^/NH_4_^+^ ratios. Increasing NO_3_^−^ proportion in the N nutrition of *E. purpurea* caused to increase in plant height and root weight considerably (Table 2).

### Essential oil analysis

The flower head, leaves, and root essential oil compositions of *E. purpurea* grown at the 50% perlite + 50% peat moss medium with perlite particle size less than 0.5 mm growing medium at different NO_3_^−^/NH_4_^+^ ratios (90:10 and 70:30) are shown in Tables 3 and 4, respectively.

**Table 3:** Essential oil chemical composition of *E.purpurea* grown at the best growing media at 90:10 NO_3_^-^/NH_4_^+^ ratio.

Components	Class ^a^	LRI ^b^	KI	Percentage		
				Flower heads	Leaves	Root
Heptane	NT	776	732.68	0.018	0.013	tr ^c^
Myrcene	MH	921	773.24	15	11	1.1
(Z)-3-Hexenol acetate	NT	930	794.80	0.32	0.25	0.11
n-Tridecene	NT	940	811.30	0.028	0.012	tr
δ-Elemene	SH	965	832.24	0.12	0.097	tr
Cyclosativene	SH	968	837.15	0.32	0.25	0.11
α-Ylangene	SH	983	858.63	0.19	0.13	0.12
α-Copaene	SH	998	906.21	1.4	1.1	0.25
α-Pinene	SH	1003	919.32	12	8.1	1.15
β-Bourbonene	SH	1010	923.68	0.22	0.19	0.064
β-Cubebene	SH	1018	969.90	0.19	0.17	0.013
β-Elemene	SH	1020	993.55	0.33	0.24	0.072
n-Tetradecene	NT	1022	1022.15	0.96	0.52	0.15
β-Caryophyllene	SH	1031	1076.38	11	7.6	1.1
β-Copaene	SH	1047	1093.50	1.1	0.78	0.15
γ-Elemene	SH	1074	1110.86	0.41	0.35	tr
trans-α-bergamotene	SH	1362	1143.80	0.78	0.43	0.096
Aromadendrene	SH	1365	1159.81	0.41	0.38	tr
α-Humulene	SHS	1370	1205.62	0.21	0.15	tr
cis-Muurola-4(14), 5- diene	SH	1390	1232.56	0.063	0.022	tr
(Z)-8-dodecen-1-ol	NT	1391	1240.25	0.014	0.027	tr
Germacrene D	SH	1412	1320.39	51	43	1.6
(E)-B-ionone	AC	1422	1329.12	0.28	0.21	tr
1-Pentadecene	NT	1430	1360.85	4.4	2.1	0.91
Bicyclogermacrene	SH	1441	1372.13	0.66	0.46	tr
α-Muurolene	SH	1468	1390.23	1.2	0.89	0.031
n-Pentadecane	NT	1476	1396.14	0.58	0.38	tr
(Z)-a-Bisabolene	SH	1479	1400.09	0.43	0.35	tr
trans-β-Guaiene	SH	1488	1423.29	0.11	0.082	tr
(E, E)-α-Farnesene	SH	1415	1439.43	0.062	0.024	tr
α-Bulnesene	SH	1520	1452.14	0.14	0.097	tr
δ-Amorphene	SH	1548	1490.33	0.19	0.85	0.053
trans-γ-Cadinene	SH	1561	1513.77	0.057	0.032	tr
δ-Cadinene	SH	1570	1521.36	0.092	0.015	tr
Selina-3,7(11)-diene	SH	1575	1525.44	0.083	0.021	tr
Germacrene B	SH	1620	1537.14	0.054	0.013	tr
Germacrene D-4-ol	OS	1630	1540.32	0.092	0.042	tr
Nerolidol acetate	OS	1662	1554.56	0.063	0.011	tr

^a^Class: *AC* apocarotenoids; *MH* monoterpene hydrocarbons; *SH* sesquiterpene hydrocarbons; *NT* non-terpenes; *OS* oxygenated sesquiterpenes.

^b^*LRI* Linear Retention Index.

^c^Trace (below 0.01%); *KI* Kovats Index.

**Table 4:** Essential oil chemical composition of *E.purpurea* grown at the best growing media at 70:30 NO_3_^-^/NH_4_^+^ ratio.

Components	Class^a^	LRI^b^	KI	Percentage		
				Flower heads	Leaves	Root
Heptane	NT	776	732.68	tr	tr	tr ^c^
Myrcene	MH	921	773.24	10	8.9	0.75
(Z)-3-Hexenol acetate	NT	930	794.80	0.21	0.11	0.083
n-Tridecene	NT	940	811.30	tr	tr	Tr
δ-Elemene	SH	965	832.24	0.082	0.023	Tr
Cyclosativene	SH	968	837.15	0.21	0.15	Tr
α-Ylangene	SH	983	858.63	0.11	0.08	0.092
α-Copaene	SH	998	906.21	0.89	0.66	0.11
α-Pinene	SH	1003	919.32	8.5	6.1	0.75
β-Bourbonene	SH	1010	923.68	0.15	0.091	Tr
β-Cubebene	SH	1018	969.90	0.12	0.024	Tr
β-Elemene	SH	1020	993.55	0.18	0.11	0.022
n-Tetradecene	NT	1022	1022.15	0.59	0.39	Tr
β-Caryophyllene	SH	1031	1076.38	7.1	5.5	0.45
β-Copaene	SH	1047	1093.50	0.84	0.62	0.083
γ-Elemene	SH	1074	1110.86	0.22	0.11	Tr
trans-α-bergamotene	SH	1362	1143.80	0.54	0.42	Tr
Aromadendrene	SH	1365	1159.81	0.21	0.15	Tr
α-Humulene	SHS	1370	1205.62	0.13	0.053	Tr
cis-Muurola-4(14), 5-diene	SH	1390	1232.56	tr	tr	Tr
(Z)-8-dodecen-1-ol	NT	1391	1240.25	tr	tr	Tr
Germacrene D	SH	1412	1320.39	47	33	0.95
(E)-B-ionone	AC	1422	1329.12	0.15	0.042	Tr
1-Pentadecene	NT	1430	1360.85	3.1	1.8	0.25
Bicyclogermacrene	SH	1441	1372.13	0.25	0.16	Tr
α-Muurolene	SH	1468	1390.23	0.87	0.64	0.034
n-Pentadecane	NT	1476	1396.14	0.18	0.091	Tr
(Z)-a-Bisabolene	SH	1479	1400.09	0.23	0.15	Tr
trans-β-Guaiene	SH	1488	1423.29	0.052	0.014	Tr
(E, E)-α-Farnesene	SH	1415	1439.43	tr	tr	Tr
α-Bulnesene	SH	1520	1452.14	0.064	0.013	Tr
δ-Amorphene	SH	1548	1490.33	0.092	0.032	Tr
trans-γ-Cadinene	SH	1561	1513.77	tr	tr	Tr
δ-Cadinene	SH	1570	1521.36	0.023	0.014	Tr
Selina-3,7(11)-diene	SH	1575	1525.44	tr	tr	Tr
Germacrene B	SH	1620	1537.14	tr	tr	Tr
Germacrene D-4-ol	OS	1630	1540.32	0.017	tr	Tr
Nerolidol acetate	OS	1662	1554.56	0.019	tr	Tr

^a^Class: *AC* apocarotenoids; *MH* monoterpene hydrocarbons; *SH* sesquiterpene hydrocarbons; *NT* non-terpenes; *OS* oxygenated sesquiterpenes.

^b^*LRI* Linear Retention Index.

^c^Trace (below 0.01%); *KI* Kovats Index.

The essential oils were separated into 51 components, 38 of them were identified, comprising 92.8% of the total essential oil yield (Tables 3 and 4). The content and composition of the essential oil exhibited a variable pattern at different plant organs at different NO_3_^−^/NH_4_^+^ ratios (Tables 3 and 4).

The most abundant terpenes including, germacrene D, myrcene, α-Pinene, β-caryophyllene, and 1-pentadecene were found in chemical composition of *E. purpurea* extract by previous researchers. Comparing of the results in present study with other researches shows the noticeable increase in essential oil composition by using novel growing media and nutrition pattern (Table 5), which is related to improve physical properties of growing media (50% perlite + 50% peat moss medium with perlite particle size less than 0.5 mm and 90:10 NO_3_^−^/NH_4_^+^ ratio).

**Table 5:** Maximum percentage of major essential oil compositions of *E.purpurea* reported in various previous studies.

	Germacrene D	Myrcene	α-Pinene	β-Caryophyllene	1-Pentadecene
	(%)				
Present study	51	15	12	11	4.4
Diraz et al. (2012)	11	–	–	7.2	–
Sitarek et al. (2017)	19	0.12	1.1	–	0.73
Thappa et al. (2003)	33	10	6.6	9.3	2.5
Nyalambisa et al. (2016)	20	–	3.7	4.5	–
Hudaib and Cavrini (2002)	29	1.7	2.3	3.1	–
Holla et al. (2005)	4.8	2.1	5.1	3.6	2.5
Kyslychenko et al. (2008)	25	11	7.5	5.2	2.9
Kan et al. (2008)	32	9.4	4.2	4.3	3.2

## Discussion

Based on open hydroponic cultivation system in the present experiment, decreasing perlite particle size, increased the retention time of nutrient solution in the culture media. Increasing nutrient accessibility for plant roots by increasing retention time improves nutrient uptake and plant growth. However, the pure perlite culture system (100% perlite, < 0.5 mm) has a very low air-filled porosity (AFP) of 33% and water holding capacity (WHC) of 56% in comparison with other fine-perlite culture media (Table 6). Accordingly, the lowest growth parameters were obtained in pure perlite medium (Table 2), which can be attributed to the rapid withdrawal of nutrient solution from the culture medium and the inability of the medium to maintain the nutrient solution. Due to the high porosity of peat mass and nutrient solution retention capability, an increase of the plant morphological parameters is expected in the presence of peat moss in various cultural media (Table 2). The noticeable increase in chlorophyll content by reducing perlite particle size implies the significant effect of culture media on photosynthesizing pigments (Table 2). It has been reported that the application of N fertilizers in the fine perlite culture media increased N content of the plants, thereby increasing their chlorophyll content, subsequently, and their ability to absorb sunlight and produce photosynthates, which resulted in their higher leaf area, and growth and yield^18,28^.

**Table 6:** Physical properties of media used in greenhouse *E. purpurea* culture.

Culture media	Water holding capacity	Air-filled porosity	Bulk density	Total porosity
	(% vol)	(%)	(g cm^−3^)	(%)
100% Pe (> 2 mm)	56	33	0.16	89
100% Pt	75	10	0.21	85
50% Pt + 50% Pe (< 0.5 mm)	68	24	0.18	92
50% Pt + 50% Pe (0.5–1 mm)	65	26	0.18	91
50% Pt + 50% Pe (1–1.5 mm)	64	28	0.18	92
50% Pt + 50% Pe (1.5–2 mm)	62	31	0.17	93
50% Pt + 50% Pe (> 2 mm)	60	33	0.17	93
30% Pt + 70% Pe (< 0.5 mm)	61	28	0.14	89
30% Pt + 70% Pe (0.5–1 mm)	58	30	0.14	88
30% Pt + 70% Pe (1–1.5 mm)	56	32	0.15	88
30% Pt + 70% Pe (1.5–2 mm)	53	33	0.15	86
30% Pt + 70% Pe (> 2 mm)	50	35	0.16	85

The numbers in the parentheses show perlite particle size.

*Pt* peat moss and *Pe* perlite.

The essential oil was characterized by a higher percentage of terpene hydrocarbons, especially the monoterpenoids, which constituted 60 to 70% of the essential oil composition. The major terpene hydrocarbons found are α-pinene, myrcene, β-caryophyllene, 1-Pentadecene, and germacrene D. The percentages of these terpene hydrocarbons were higher in flower head than leave and root at both NO_3_^−^/NH_4_^+^ ratios. The most abundant terpene found in the essential oil was germacrene D, which showed a remarkable rise from 1.5% in root to 51% in flower head and 0.95% in root to 47% in flower head at 90:10 and 70:30 NO_3_^−^/NH_4_^+^ ratios, respectively. Variability was also obtained in the concentration of other compositions. The results (Tables 3 and 4) indicate that the various components of the essential oil of *E. purpurea* are specific to the plant organs, which influence their concentration.

The variations in the concentrations of various essential oil compositions at different NO_3_^−^/NH_4_^+^ ratios (Tables 3 and 4) may be due to supply different amounts of NO_3_^−^ to the plant. The presence of N as a key factor can affect the production of essential oils in aromatic plants^29^. Nitrogen is critical factor in biosynthesis pathway of essential oil in medicinal and aromatic plants^30^. Nitrogen increases photosynthetic efficiency and plays an important role in increasing the amount of essential oil by increasing the number and area of leave and providing a suitable condition for receiving sunlight energy and also participating in the structure of chlorophyll and enzymes involved in photosynthetic carbon metabolism^31^. Nitrogen is an essential nutrient in plants used to synthesize many organic compounds in plants such as nucleic acids, enzymes, proteins, and amino acids, which are necessary for essential oil biosynthesis pathway^32^. Besides, essential oils are terpenoids compounds whose constituent units (isonoids) such as isopentenyl pyrophosphate and dimethyl ally pyrophosphate are strongly formed into adenosine triphosphate (ATP) and nicotinamide adenine dinucleotide phosphate (NADPH), and due to the effect of N in the production of these compounds, the amount of essential oil increased^33^. Nitrogen increases the essential oil content of plants by increasing the dry weight (Nyalambisa et al. 2016). Comparing of the results in Tables 3 and 4 indicated that increase of NO_3_^−^ concentration could increase the percentage of essential oil composition due to its effect on essential oil biosynthesis as demonstrated in previous researches^34^.

Germacrene D, myrcene, α-Pinene, β-Caryophyllene, and 1-Pentadecene were the major compositions of essential oil of *E.purpurea* grown in very fine-grade (< 0.5 mm) perlite with 50:50 v/v perlite to peat moss ratio (Tables 3 and 4). The compositions have a valuable beneficial effects in medicine and agriculture industries^7^.

Germacrene D is a natural hydrocarbon, belongs to sesquiterpenes, which is found in aromatic plants^27^. The hydrocarbon is a useful bioactive phytochemical compound in human health Maintains healthy blood pressure is one of the important roles of germacrene compounds in humans^8^. The antimicrobial properties of germacrene D were reported in previous researches^10^. Anti-inflammatory, antimicrobial, and antioxidant effects of germacrene D are also well known^8^. The anti-insect influence of germacrene D has been reported in previous studies^10^. Myrcene is a terpene with anti-inflammatory and anti-depressant effects^14^. Regulating the efficiency of other terpenes and cannabinoids by increasing of myrcene is recognized previously^7^. Pinene has a several of potential benefits, including anti-inflammatory, antimicrobial, antitumor, antioxidant, and neuroprotective effects. It may also help counteract the short-term memory issues that many people experience. Beta-caryophyllene is also known for antioxidant and anti-inflammatory medicinal effects. It is especially useful to decrease pain and anxiety^35^.

It was found that the mixture of peat moss into very fine-grade perlite (< 0.5 mm) at 50:50 v/v perlite to peat moss ratio had a significant increase in plant growth parameters, which increased by increasing of NO_3_^−^/NH_4_^+^ ratio. The essential oil content was significantly highest in the 50:50 v/v perlite to peat moss ratio (perlite particle size less than 0.5 mm) than others. The major terpene hydrocarbons found in extract of *E. purpurea* with the best growth parameters were germacrene D, myrcene, α-pinene, β-caryophyllene, and 1-Pentadecene, respectively. The percentages of these terpene hydrocarbons were increased by increasing of NO_3_^−^/NH_4_^+^ ratio. Using of perlite and peat moss mixture for plant cultivation not only affects the plant growth parameters and essential oil compositions, but also reduces production costs in hydroponic systems.
